# Flying electron spin control gates

**DOI:** 10.1038/s41467-022-32807-x

**Published:** 2022-09-14

**Authors:** Paul L. J. Helgers, James A. H. Stotz, Haruki Sanada, Yoji Kunihashi, Klaus Biermann, Paulo V. Santos

**Affiliations:** 1 Paul-Drude-Institut für Festkörperelektronik, Leibniz-Institut im Forschungsverbund Berlin e.V., Hausvogteiplatz 5-7, 10117 Berlin, Germany; 2 NTT Basic Research Laboratories, NTT Corporation, 3-1 Morinosato-Wakamiya, Atsugi, Kanagawa 243-0198 Japan; 3 Department of Physics, Engineering Physics & Astronomy, Queen’s University, Kingston, ON K7L 3N6 Canada

**Keywords:** Spintronics, Electronic and spintronic devices, Nanowires, Nanowires, Electronic properties and materials

## Abstract

The control of "flying” (or moving) spin qubits is an important functionality for the manipulation and exchange of quantum information between remote locations on a chip. Typically, gates based on electric or magnetic fields provide the necessary perturbation for their control either globally or at well-defined locations. Here, we demonstrate the dynamic control of moving electron spins via contactless gates that move together with the spins. The concept is realized using electron spins trapped and transported by moving potential dots defined by a surface acoustic wave (SAW). The SAW strain at the electron trapping site, which is set by the SAW amplitude, acts as a contactless, tunable gate that controls the precession frequency of the flying spins via the spin-orbit interaction. We show that the degree of precession control in moving dots exceeds previously reported results for unconstrained transport by an order of magnitude and is well accounted for by a theoretical model for the strain contribution to the spin-orbit interaction. This flying spin gate permits the realization of an acoustically driven optical polarization modulator based on electron spin transport, a key element for on-chip spin information processing with a photonic interface.

## Abstract (web summary)

Spin qubits are a platform for quantum computing. There are many advantages for quantum information processing if the spin qubit can move. Here, Helgers et al. use a surface acoustic wave to define a moving quantum dot and demonstrate the magneticfield-free control of the spin precession, bringing “flying” spin qubits a step closer.

## Introduction

The spin field-effect transistor proposed by Datta and Das^1^ relies on the precession of moving (or flying) electron spins around the effective magnetic field ${\overrightarrow{B}}_{{{{{{{{\rm{SO}}}}}}}}}({{{{{{{\bf{k}}}}}}}})$ associated with the spin-orbit (SO) interaction, which depends on electron momentum *ℏ***k**. ${\overrightarrow{B}}_{{{{{{{{\rm{SO}}}}}}}}}$ can be electrically controlled by an electrostatic gate via the Bychkov-Rashba effect^2^, thus opening the way for dynamic spin manipulation by electric fields. Spin transistors based on the electrical spin control have so far been demonstrated only for ballistic spin transport along short (<2 μm) channels^3,4^.

SO-based spin control over long transport distances, which normally takes place in the diffusive regime, faces two main challenges. The first is D’yakonov-Perel’ (DP) spin dephasing^5^ associated with the momentum-dependence of ${\overrightarrow{B}}_{{{{{{{{\rm{SO}}}}}}}}}$. Approaches to reduce DP spin dephasing and enable long-range spin transport lengths (*ℓ*_*s*_) include the engineering the SO interaction^6–11^ as well as exploitation of motional narrowing effects^5^. The latter takes advantage of the inverse dependence of the DP dephasing rate on the carrier scattering time, which can be achieved via increased momentum scattering by impurities^12^ or at the boundaries of narrow transport channels (i.e., channel widths less than the precession period, *L*_SO_ under ${\overrightarrow{B}}_{{{{{{{{\rm{SO}}}}}}}}}$). This latter approach has been realized using quantum wire channels^13,14^ as well as by enclosing the spins within moving potential dots^15,16^.

**Fig. 1:**
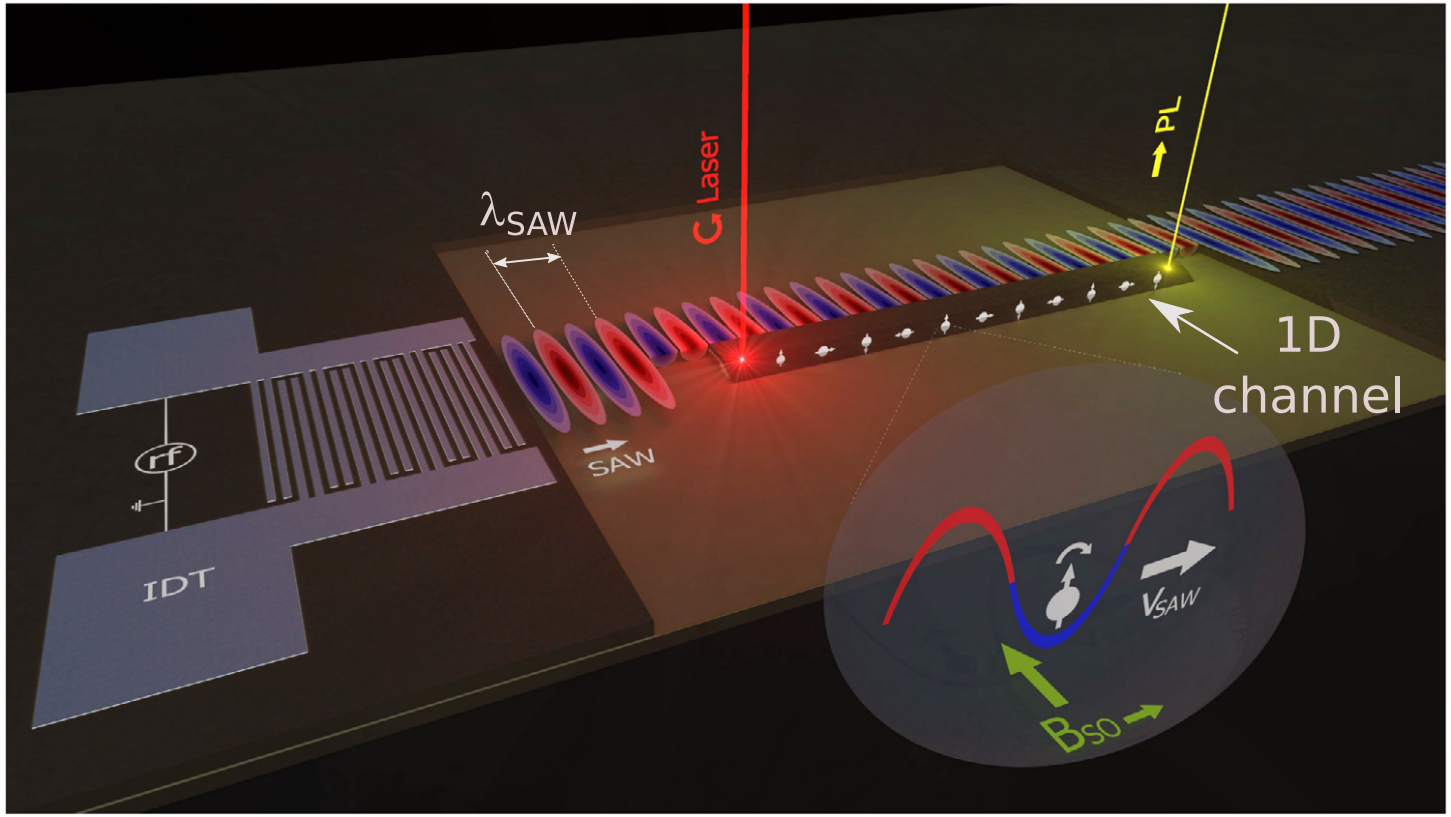
Flying control gate for electron spins. A surface acoustic wave (SAW) excited by an interdigital acoustic transducer (IDT) is applied along a quasi-one dimensional (1D) semiconductor channel. The piezoelectric potential of the SAW creates moving, quasi-zero dimensional (0D) potential dots, which capture spin-polarized electrons and holes excited by a circularly polarized laser beam and transport them along the channel with the SAW velocity. Simultaneously, the SAW strain field induces an effective spin-orbit magnetic field *B*_*SO*_ at the carrier transport sites with amplitude proportional to the SAW field. The latter acts as a flying spin gate to control the spin precession rate.

The second major challenge for spin control is to devise field configurations to drive spin motion and, simultaneously, generate a tunable ${\overrightarrow{B}}_{{{{{{{{\rm{SO}}}}}}}}}$ for controlled spin precession. An elegant solution is offered by the carrier transport by moving potential dots produced by a surface acoustic wave (SAW) along a one-dimensional (1D) channel. Figure 1 depicts an example based on a quantum wire (QWR) transport channel. Here, the moving piezoelectric potential modulation produced by the SAW stores spin-polarized electrons and holes excited by a circularly polarized optical beam at different SAW subcycles and transports them with the acoustic velocity. The spatial separation of electrons and holes prevents recombination^17^ and, simultaneously, also suppresses spin relaxation due to the electron-hole exchange interaction^18^. The ambipolar SAW transport can thus transfer electron spins over long distances (up to 100 μm) while enabling optical spin readout by detecting the polarization of photons emitted by the recombination of the transported carriers^16,19–22^.

Concomitantly with the transport, the SAW strain^23–27^ and piezoelectric fields at the carrier location induce a ${\overrightarrow{B}}_{{{{{{{{\rm{SO}}}}}}}}}$ contribution, which moves congruently with the carriers [cf. Fig. 1]. The SAW amplitude acts, therefore, as a contactless, flying spin gate, which dynamically controls the rate of spin precession during transport. In this work, we demonstrate that long-range acoustic transport can be combined with a high degree of dynamic control of the spin precession rate (by over 250%) if the flying spins are transported while confined within micron-sized moving potential dots. This degree of precession control exceeds by over an order of magnitude previous results for acoustic transport in 2D quantum well (QW) channels^21,28^. We also show that the precession rate is mainly mediated by the acoustic strain imparted by the SAW in the storage phase of the electron spins during transport. The latter essentially enables the carrier wave to also act as a flying spin gate controlled by the SAW amplitude.

The study addresses two types of acoustically defined, moving potential dots. In one system, the dots are formed by propagating a SAW along a quasi-planar GaAs QWR [as illustrated in Fig. 1]. We have also investigated spin transport and manipulation in moving potential dots created by the interfering piezoelectric fields of orthogonal SAW beams (denoted as dynamic quantum dots, DQDs^15,29^). For both types of moving dots, we experimentally demonstrate flying spin gates using optically excited spins that can be acoustically transported over large distances (tens of microns) with the spin precession rate controlled over a wide dynamic range by the SAW amplitude (Section: Results) and, notably, without external electric or magnetic fields. The measured precession rates are well accounted for by an analytical model for the SO fields generated by the SAW strain and piezoelectric fields, from which the strain-related SO parameters can be experimentally determined (Section: SAW-related spin-orbit fields). As a further check of consistency, we show that spin precession rates and their dependence on the acoustic fields are also in good agreement with microscopic calculations of the spin splittings under the SAW field using a tight-binding approach. Interestingly, while the moving dot geometry enables precession control, a substantial enhancement of the spin lifetime due to motional narrowing is only observed for the DQDs. The limited spin lifetimes in the QWRs, in contrast, is attributed to the fact that the positive impact of lateral confinement on the spin lifetime is offset by spin scattering at the lateral, compositional interfaces. Even so, the dramatic ability of the flying spin gates to control the precession frequency by the SAW amplitude demonstrates a processor for optically encoded polarization information based on the dynamic control of electron spins during acoustic transport.

## Results

### Acoustic spin transport

**Fig. 2:**
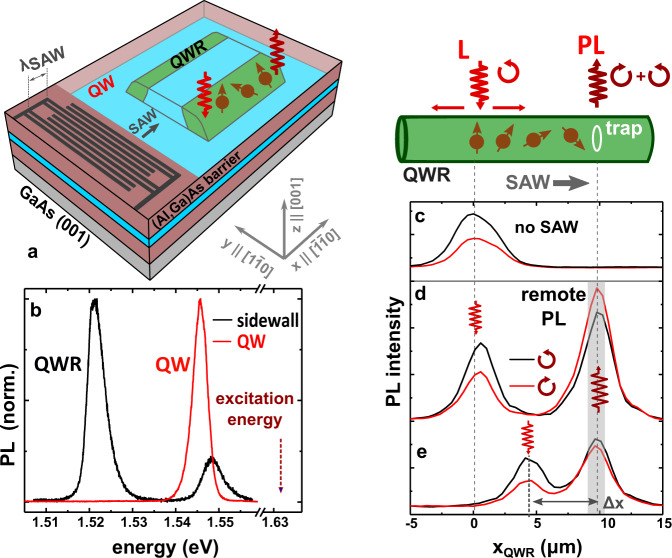
Optically detected transport of spins in planar quantum wires. **a** Sidewall quantum wires (QWRs) formed by the epitaxial overgrowth of a quantum well (QW) on a GaAs (001) substrate structured with shallow ridges^30^. The QWRs are 200 nm-wide and have a length defined by photolithography, which is typically several tens of microns long. Photoexcited carriers are transported along the QWR by surface acoustic waves (SAWs) generated by interdigital acoustic transducers (IDTs). **b** Photoluminescence (PL) spectra recorded outside (red) and on (black) a ridge sidewall showing the emission lines of the QW (1.548 eV) and QWR (1.521 eV), respectively. **c**–**e** Profiles of the right (black, ${I}_{PL}^{\circlearrowleft}$) and left (red, ${I}_{PL}^{\circlearrowright}$) circularly polarized PL along the QWR axis (*x*_QWR_ coordinate) recorded under the configuration illustrated in the upper panel (**c**) in the absence and (**d**, **e**) under a SAW. The PL was excited by a right-circularly polarized laser spot focused at *x*_QWR_ = 0 for (**c**) and (**d**), and at *x*_QWR_ = 4.5 μm for (**e**).

The structure of the QWR samples is illustrated in Fig. 2a. As described in the Methods section, the GaAs QWRs are fabricated by combining steps of surface patterning and overgrowth by molecular beam epitaxy (MBE). In this process, the growth of an (Al,Ga)As/GaAs/(Al,Ga)As QW stack over a patterned ridge leads to the formation of a thicker GaAs region (the QWR) at the ridge sidewalls, which is electrically connected to the QW^30^. The photoluminescence (PL) features of the sample are summarized in Fig. 2b. Here, the red and black curves compare PL spectra recorded under confocal excitation and detection on the QW region and on the ridge sidewall (corresponding to the QWR position), respectively. The former shows a single PL line at 1.546 eV with a linewidth (full-width-at-half-maximum, FWHM) of 4 meV associated with the electron-heavy hole QW exciton. The spectrum recorded on the ridge sidewall shows the excitonic emission from the QWR at 1.521 eV with a FWHM of 4.6 meV together with a second line at 1.548 eV (FWHM of 5.4 meV). The latter stems from the QW regions near the QWR, which are slightly thinner than those further from the ridge sidewalls^30^. The energy difference between the two lower lying lines yields a lateral confinement energy for electrons (holes) in the QWR of approximately 22 meV (4 meV).

The optical detection of acoustically driven spin transport along the QWR is illustrated in Fig. 2c–e. The experiments were carried out using the geometry depicted in the upper right panel by exciting spins using a right-hand circularly polarized laser beam with energy below the QW resonance. The black and red profiles display the spatial distribution of the integrated PL from the QWR [detection window from 1.517 eV to 1.526 eV, cf. Fig. 2a] with right- (${I}_{PL}^{\circlearrowleft }$) and left-hand (${I}_{PL}^{\circlearrowright }$) circular polarizations, respectively. In the absence of a SAW [Fig. 2c], the emission is restricted to the regions around the excitation spot and has a net right-hand circular polarization. Since hole-spin relaxation is typically much faster than the one for electrons^31^, the PL polarization ${\rho }_{s}=({I}_{PL}^{\circlearrowleft }-{I}_{PL}^{\circlearrowright })/({I}_{PL}^{\circlearrowleft }+{I}_{PL}^{\circlearrowright })$ essentially reflects the electron spin dynamics. The PL profiles can be fitted with a Gaussian characterized by a FWHM of 4 μm. The increased spatial PL spread compared to the size of the laser spot is attributed to the diffusion of the spin polarized carriers along the QWR axis.

Figure 2d, e displays the corresponding profiles acquired in the presence of a SAW that captures the spin polarized carriers and transports them to trap centers at *x*_QWR_ = 10 μm, where the electrons and holes recombine. The trapping and recombination centers are defect regions along the QWR^30^. The trapping mechanism, which leads to emission at the same energy as the QWR, is attributed to carrier capture at centers at the interface between the QW (or QWR) layer and the (Al,Ga)As barrier layers assisted by the transverse component of the SAW piezoelectric field, *F*_*z*_^30,32^. These centers can capture carriers of one polarity during one half-cycle of the SAW and release them during the passage of carriers of the opposite polarity in the subsequent SAW half-cycle when *F*_*z*_ reverses its sign. The centers provide efficient recombination centers to stop the transport and monitor the PL polarization. Note that the PL polarization depends on the transport distance Δ*x* changing from right- [Fig. 2e] to left-hand circular polarization [Fig. 2d] as Δ*x* increases from 5 to 10 μm. This is due to the larger precession angle under the SO field accumulated while traveling a longer distance.

### Spin precession control

**Fig. 3:**
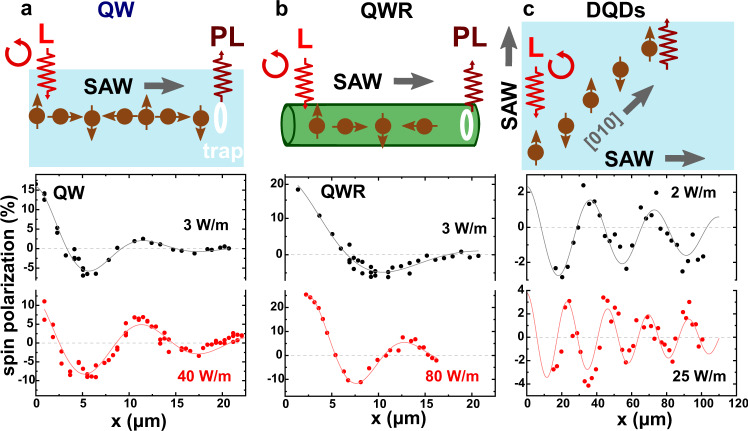
Acoustic control of the precession rate of moving spins. Spin polarization, *ρ*_*s*_, during transport along (**a**) a quantum well (QW, excitation wavelength *λ*_*L*_ = 776 nm and power *P*_*exc*_ = 15 μW, 20 K), (**b**) a quantum wire (QWR, *λ*_*L*_ = 776 nm, *P*_*exc*_ = 30 μW (3 W m^−1^) and *P*_*exc*_ = 150 μW (80 Wm^−1^), 20 K), and (**c**) dynamic dots (DQDs, *λ*_*L*_ = 776 nm, 12 K) . The upper panels illustrate the experimental setup: spin-polarized electrons (brown) are optically injected by a circularly polarized laser (*L*, red) and transported by a SAW along the [110]-direction (QW and QWR) or along the [010]-direction (DQDs). The curves correspond to different SAW linear power densities, *P*_SAW_.

The procedure depicted in Fig. 2c–e was applied to determine the spatial dependence of the spin polarization *ρ*_*s*_ on the SAW amplitude. Figure 3 summarizes *ρ*_*s*_ profiles for acoustic transport for the three geometries illustrated in the corresponding upper panels: (a) along the QW, (b) along the QWR and (c) using DQDs. For the QW and QWR, the transport distance *x* is along the [110]-direction, but it is along the [010]-direction for the DQDs. In all cases, *ρ*_*s*_ oscillates with a period that reduces with increasing SAW amplitudes thus demonstrating the operation principle of the acoustic spin gate. In particular, a distinct reversal of the spin polarization can be observed for both the QWR (*x* = 12.5 μm) and DQD (*x* = 25 μm) geometries, with the latter occurring well within the spin coherence length of transport.

The solid lines are fits of the experimental data to an exponentially decaying cosine function of the form

1 $${\rho }_{s}(x)={\rho }_{s}(0)\cos \left(\frac{{\Omega }_{SO}}{{v}_{SAW}}\Delta x\right){e}^{-\Delta x/{\ell }_{s}}.$$

**Fig. 4:**
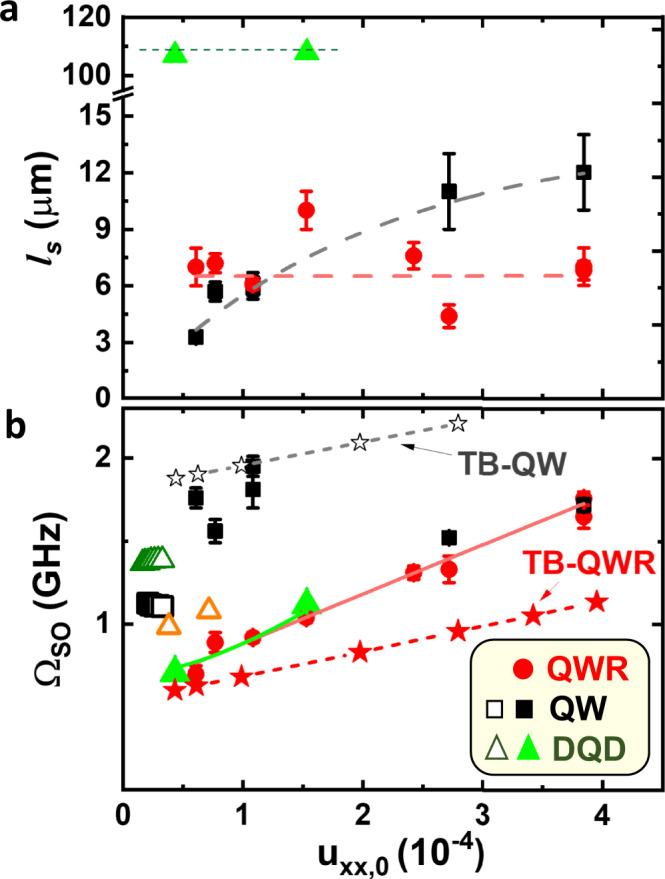
Spin dynamics under acoustic fields. **a** Spin transport length, *ℓ*_*s*_, and **b** angular precession frequency, Ω_SO_, as a function of the strain amplitude *u*_*xx*,0_ for transport along the QW (10 nm thick, black solid squares), QWR (red solid dots), and DQDs (30 nm thick QW, green solid triangles) investigated in this work. The error bars represent the standard deviation in determining the fitted parameters. The solid red line in (**b**) is a fit of the QWR data to Eq. (2); the green curve superimposed on the solid triangles is the prediction of Eq. (3) for the DQDs using the *C*_3_ parameter determined from the fit. Note that for the DQDs, the horizontal axis is the strain amplitude per acoustic beam and the Ω_SO_ values were scaled by a factor of $1/\sqrt{2}$ to account for the higher transport velocity as compared to the QW or QWR (cf. Eqs. (2) and (3)). The open and solid ⋆'s yield the Ω_SO_ obtained from tight-binding (TB) calculations for the studied QWs (TB-QW) and QWRs (TB-QWR), respectively. The dashed lines in (**a**) are guides to the eye. The open symbols in (**b**) represent previously reported data for spin transport by a single SAW^21^ (series of open squares at *u*_*xx*_ = 0.2 × 10^−4^), DQDs in a 20 nm thick QW^21^ (series of open triangles at *u*_*xx*_ = 0.2 × 10^−4^), as well as for DQDs in a 20 nm-thick QW^29^ (orange triangle).

The fit parameters are the total SO angular precession frequency Ω_SO_ as well as the characteristic spin transport length *ℓ*_*s*_. Ω_SO_ can be expressed in terms of the oscillation period *L*_*SO*_ = 2*πv*_SAW_/Ω_*SO*_. The values of *ℓ*_*s*_ and Ω_SO_ obtained from the fits are displayed as filled symbols in Fig. 4 as a function of the SAW amplitude (stated in terms of the amplitude of the uniaxial strain component ${u}_{xx,0}\propto \sqrt{{P}_{{{{{{{{\rm{SAW}}}}}}}}}}$, where *P*_SAW_ is the SAW linear power density defined as the ratio between the acoustic power and the SAW beam width). For the QWR (solid red dots), *ℓ*_*s*_ ~ 7 μm is independent of the SAW amplitude, and this value is equal to the one measured in the absence of a SAW (see Supplementary Information Section SM3). In contrast, *ℓ*_*s*_ for the QW (solid black squares) increases with SAW power [cf. Fig. 4a]–a behavior which will be further addressed below. For the DQDs (solid green triangles), *ℓ* is considerably larger and comparable to the maximum measured transport distance.

The open symbols in Fig. 4b display, for comparison, values for Ω_SO_ in QWs and DQDs reported in previous studies, which cover a much narrower range of acoustic amplitudes than the new results represented by filled symbols presented here. A remarkable finding is the wide degree of control of Ω_SO_ by the SAW amplitude for transport both along the QWRs and by DQDs. In these structures, Ω_SO_ increases by as much as 220% and 70% with increasing SAW amplitude, respectively. These ranges of precession control far exceed those in previous reports, where the SAW-induced changes are limited to typically less than 10%. The QWRs and DQDs thus act as tunable spin logic and polarization modulators, where the tunability is provided by the acoustic amplitude. Furthermore, the strong Ω_SO_ dependence on the acoustic amplitude in QWRs and DQDs contrasts with the one in the QWs studied here (solid squares), where Ω_SO_ remains within a range of± 15% with no clear trend with increasing SAW amplitude.

### SAW-related spin-orbit fields

**Fig. 5:**
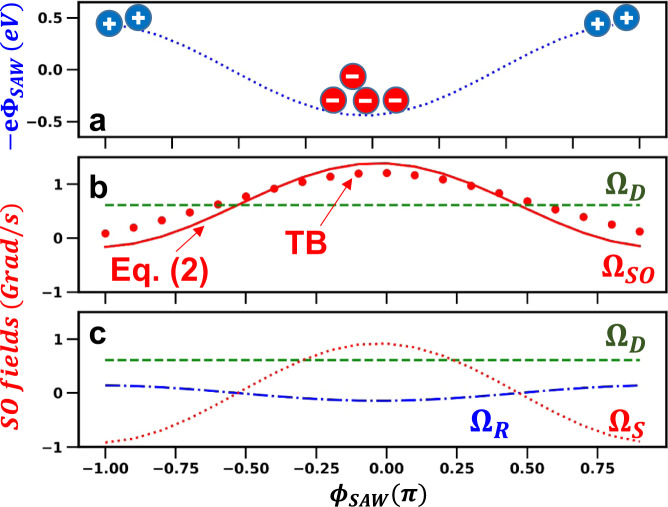
Tight-binding calculations of the spin precession rate. SAW phase (*ϕ*_SAW_) dependence of the (**a**) electronic piezoelectric energy ( − *e*Φ_SAW_, dotted blue line) and of the (**b**) electron-spin precession frequency determined by the tight-binding (TB) approach for a QW with the same thickness as the QWR under *P*_SAW_ = 100 W/m (dots). The solid red line yields the total SO field Ω_SO_ obtained from the analytical approximation of Eq. (2). Ω_D_ (dashed green line) is the Dresselhaus contribution. **c** The green dashed (Ω_D_), dotted red (Ω_S_), and dot-dashed blue (Ω_R_) curves are the corresponding Dresselhaus, strain, and piezoelectrically induced Rashba contributions, respectively.

In order to quantify the SO fields induced by the SAW, we first note that the strain field of a Rayleigh SAW along $\tilde{{{{{{{{\bf{x}}}}}}}}}|\vert [110]$ consists of two uniaxial strain components ${u}_{xx}={u}_{xx,0}\cos ({\phi }_{{{{{{{{\rm{SAW}}}}}}}}})$ and ${u}_{zz}={u}_{zz,0}\cos ({\phi }_{{{{{{{{\rm{SAW}}}}}}}}})$ as well as a phase-shifted shear component ${u}_{xz}={u}_{xz,0}\sin ({\phi }_{{{{{{{{\rm{SAW}}}}}}}}})$^33^. Here, $\tilde{{{{{{{{\bf{z}}}}}}}}}|\vert [001]$ is the QW growth axis, *ϕ*_SAW_ = (*k*_SAW_*x* − *ω*_SAW_*t*) is the SAW phase, and *k*_SAW_ = 2*π*/*λ*_SAW_ and *ω*_SAW_ are the SAW wave vector and angular frequency, respectively. During transport, the SAW piezoelectric potential ${\Phi }_{{{{{{{{\rm{SAW}}}}}}}}}={\Phi }_{SAW,0}\cos ({\phi }_{{{{{{{{\rm{SAW}}}}}}}}})$ captures electrons around the SAW phase *ϕ*_SAW_ = 0 corresponding to the minimum piezoelectric energy, as illustrated in Fig. 5a. In the following analysis, we will assume that the electron spins remain stored around these phases during the transport, where the transverse piezoelectric field *F*_*z*_ also attains its maximum value (see Supplementary Information Section SM6).

The lateral dimensions of the QWRs and DQDs are much larger than the QWR (or QW) thickness. For the determination of the Ω_SO_ in QWRs and DQDs, we will neglect the role of the lateral confinement (and of the lateral interfaces) and assume that Ω_SO_ is essentially equal to the one in a QW of the same thickness. We now address the different SO mechanisms acting on spins acoustically transported along the *x*-direction of a QW with the SAW velocity *v*_SAW_. The effective electron wave vector *k*_*x*_ = m^*^v_SAW_/*ℏ* is determined by the effective electron mass m^*^ (*ℏ* is the reduced Planck’s constant) (see Supplementary Information Section SM4). The bulk inversion asymmetry (BIA) of the III-V lattice induces an intrinsic SO field (the Dresselhaus term^34^), which leads to the precession of the moving spins with an angular frequency $\hslash {\Omega }_{{{{{{{{\rm{D}}}}}}}}} \sim \gamma {k}_{x}{\left(\pi /{w}_{z}\right)}^{2}$. Here, *w*_*z*_ denotes the extension of the electron wave function along *z*. The structural inversion asymmetry (SIA) induced by the SAW gives rise to precession components related to the strain field, $\hslash {\Omega }_{{{{{{{{\rm{S}}}}}}}}}=\frac{1}{2}{C}_{3}{u}_{xx}{k}_{x}$^20^, as well as to the piezoelectric field *F*_*z*_, *ℏ*Ω_R_ = 2*r*_41_*F*_*z*_*k*_*x*_. The terms *γ*, *r*_41_, and *C*_3_ are material parameters quantifying the strength of the different SO contributions (cf. Supplementary Information Section SM4). The previous expressions can be combined such that the spin precession frequency amplitude for transport by a single SAW beam (Ω_SO_) and by DQDs (${\Omega }_{{{{{{{{\rm{SO}}}}}}}}}^{(DQD)}$) can be stated as^20,21^:

2 $${\Omega }_{{{{{{{{\rm{SO}}}}}}}}}={\Omega }_{{{{{{{{\rm{D}}}}}}}}}\hat{{{{{{{{\bf{y}}}}}}}}}-({\Omega }_{{{{{{{{\rm{R}}}}}}}}}+{\Omega }_{{{{{{{{\rm{S}}}}}}}}})\hat{{{{{{{{\bf{y}}}}}}}}}$$

3 $${\Omega }_{{{{{{{{\rm{SO}}}}}}}}}^{(DQD)}=\sqrt{2}{\Omega }_{{{{{{{{\rm{D}}}}}}}}}{\hat{{{{{{{{\bf{x}}}}}}}}}}^{\prime}-2({\Omega }_{{{{{{{{\rm{R}}}}}}}}}+{\Omega }_{{{{{{{{\rm{S}}}}}}}}}){\hat{{{{{{{{\bf{y}}}}}}}}}}^{\prime}$$

In Eq. (3), $\hat{{{\bf{x}}}}^{\prime}={{\bf{x}}}-{{\bf{y}}}$ and $\hat{{{\bf{y}}}}^{\prime}={{\bf{x}}}+{{\bf{y}}}$ are the unit vectors parallel and perpendicular to the DQD propagation while Ω_R_ and Ω_S_ refer to precession frequencies for a single SAW beam along $\hat{{{{{{{{\bf{x}}}}}}}}}$. The $\sqrt{2}$ term in second equation arises from the fact that the propagation velocity of the DQDs equals to the vector sum of the velocities of the individual SAW beams. Note that while the SAW-dependent fields Ω_R_ and Ω_S_ are colinear to the BIA contribution Ω_D_ for acoustic transport along the $\hat{{{{{{{{\bf{x}}}}}}}}}$-direction in the QW and QWR, they are orthogonal to Ω_D_ for transport by DQDs.

Since *F*_*z*_ ∝ *u*_*xx*_ (and, hence, Ω_R_ ∝ Ω_S_), Eq. (2) predicts a linear dependence of Ω_SO_ on the SAW amplitude. For a quantitative comparison, we first note that while *γ* = (17 ± 2) × 10^−30^ eVm^3^ ^29^ and *r*_41_ = − (59 ± 7) × 10^−21^ em^2^ ^20^ have well-established values, the reported values of the strain-related parameter *C*_3_ span a wide range (e.g., from 0.81 eVnm in n-type doped bulk GaAs^24,26^ to 0.31 eVnm in a GaAs(001) QW^27^). The red solid line in Fig. 4b is a fit of Eq. (2) to our QWR data (filled symbols), from which we determine *C*_3_ = ( − 2.6 ± 0.1) eVnm (details in Supplementary Information Section SM6). While larger than previously reported values, this *C*_3_ value also reproduces very well the measured data for DQDs, as indicated by the green line in the figure. The good quality of the fit also supports the assumption that the electrons remain around the phase *ϕ*_SAW_ = 0 during transport.

The solid line in Fig. 5b displays the dependence of Ω_SO_ on SAW phase (*ϕ*_SAW_) as predicted by Eq. (2) for a QWR under *P*_SAW_ = 100 W/m. The different contributions Ω_D_, Ω_S_, and Ω_R_ are compared in Fig. 5c. The total spin-orbit field Ω_SO_ oscillates around an average value given by Ω_*D*_ following the harmonic dependence of the SAW-related fields Ω_*S*_ and Ω_*R*_, which have opposite signs. The amplitude of the strain-related contribution ∣Ω_*S*_∣ is much larger than the one due to the piezoelectric field (i.e., Ω_*R*_) and also exceeds the phase-independent Dresselhaus contribution Ω_*D*_.

### Tight-binding calculations of the spin splittings

In order to corroborate the predictions of the analytical model, we have also calculated the SO field Ω_SO_ in QWs using the tight-binding (TB) method^35,36^. In the TB calculations, the effects of the lateral confinement were neglected and the QWRs modeled as a thick QW (i.e., with a thickness equal to the one of the QWR used in the experiments). The dots in Fig. 5b compare the TB predictions for the Ω_SO_ dependence on *ϕ*_SAW_ in a QWR with the analytical model of the previous section (i.e., Eq. (2)). The two procedures give an oscillatory behavior for Ω_SO_ with a sligthly smaller modulation amplitude for the TB case.

To compare the TB results with the experiments, we again assume that the electrons are stored around the phases *ϕ*_SAW_ = 0 of minimum electronic piezoelectric energy − *e*Φ_SAW_ [cf. Fig. 5a]. The SAW amplitude dependence of Ω_SO_ at these phases as determined by the TB method for different SAW amplitudes is displayed by the ⋆’s connected by dashed lines in Fig. 4b for the QWR (curve labeled as TB-QWR) and QW (TB-QW). The two lines have the same slope since the strain and electric field related contributions to Ω_SO_ do not depend on the thickness of the structures. They are vertically displaced due to the larger Dresselhaus contribution for the QW structure with narrower QWs. The sign of the effective *C*_3_ parameter determined by the TB method agrees with the experiments, thus reproducing the increase of the precession rate with SAW amplitude. Its magnitude ∣*C*_3_∣ = 1.65 eVnm is slightly (33%) smaller than the experimentally determined one, but still well above previously published experimental values.

### Dimensionality effects on the spin dynamics

**Fig. 6:**
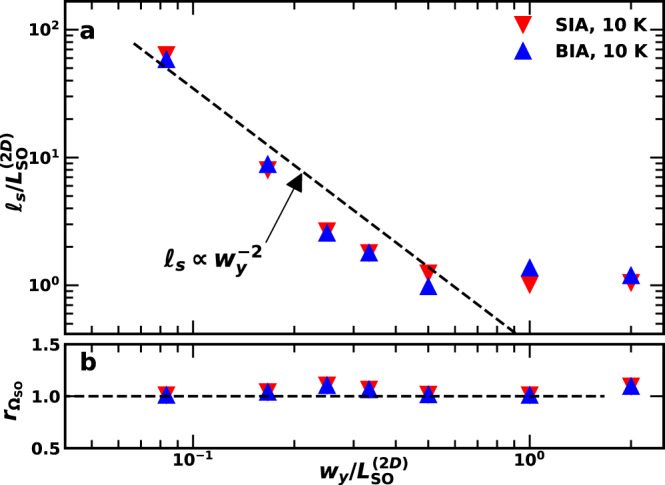
Dimensionality effects on spin dephasing. **a** Spin transport length, *ℓ*_*s*_, and (**b**) precession frequency ratio ${r}_{{\Omega }_{{{{{{{{\rm{SO}}}}}}}}}}={\Omega }_{{{{{{{{\rm{SO}}}}}}}}}({w}_{y})/{\Omega }_{{{{{{{{\rm{SO}}}}}}}}}({w}_{y}\to \infty )$ as a function of the channel width, *w*_*y*_, as determined from Monte-Carlo simulations of the acoustic spin transport under SO fields with BIA and SIA symmetries. The dashed line in **a** sketches the motional narrowing prediction (see text for details). The simulations were carried out assuming a mobility μ = 4 m^2^/(Vs) and temperature *T* = 10 K.

One interesting observation in connection with Figs. 3 and 4a is that the spin transport lengths in the QWRs are much shorter than the ones in the DQDs, despite the much smaller lateral confinement dimensions in the former structures. To understand the impact of the lateral confinement and motional narrowing effects on the spin dynamics, we have carried out Monte-Carlo simulations of the acoustic spin transport for channels with different widths *w*_*y*_ (for details, see Supplementary Information Section SM7). The results are summarized in Fig. 6. As expected from motional narrowing in the regime of 1D spin transport (i.e., for ${w}_{y} \, < \, {L}_{{{{{{{{\rm{SO}}}}}}}}}^{(2{{{{{{{\rm{D}}}}}}}})}$), *ℓ*_*s*_ increases with decreasing channel width as ${\ell }_{s}\propto {w}^{-{n}_{w}}$ with *n*_*w*_ = 2 ^13^. This dependence of *ℓ*_*s*_ applies to both BIA and SIA SO fields.

Assuming ${L}_{{{{{{{{\rm{SO}}}}}}}}}^{(2{{{{{{{\rm{D}}}}}}}})}={\ell }_{s} \sim 12\,\mu$m (as for the QW in Fig. 4), the previous expressions predicts an increase of *ℓ*_*s*_ by a factor of ${(2{L}_{{{{{{{{\rm{SO}}}}}}}}}^{(2{{{{{{{\rm{D}}}}}}}})}/{\lambda }_{{{{{{{{\rm{SAW}}}}}}}}})}^{2} \sim 18$ for electron spins enclosed in DQDs defined by SAW beams with a wavelength *λ*_SAW_ = 5.6 μm. Such an increase qualitatively explains the long spin transport lengths measured for DQDs. The same expression also yields huge spin lifetimes and transport lengths for the much narrower QWRs, which are, however, not observed in the experiments. A possible explanation lies on the nature of the lateral confinement potential: while purely electrostatic for DQDs, it is imposed by structural interfaces in the QWRs. Spin dephasing due to frequent scattering events at these interfaces (known as Elliot-Yafet processes^37^), as previously postulated for QWRs^14,38^, as well as interface-related SO fields may thus limit *ℓ*_*s*_ in structural channels.

A further remarkable experimental result is the weak dependence of the spin precession rate Ω_SO_ on SAW amplitude in QW structures as compared to QWRs and DQDs [cf. Figs. 3a and 4b]. This behavior also contrasts with the TB predictions as well as with the model leading to Eq. (2), which predicts the same linear dependence Ω_SO_ for both QWR and QW structures. Furthermore, it is at odds with the Monte-Carlo simulations illustrated in Fig. 6b, which yield the dependence of the spin precession frequency on channel width. Here, ${r}_{{\Omega }_{{{{{{{{\rm{SO}}}}}}}}}}={\Omega }_{{{{{{{{\rm{SO}}}}}}}}}({w}_{y})/{\Omega }_{{{{{{{{\rm{SO}}}}}}}}}({w}_{y}\to \infty )$ is the ratio between the precession frequencies in a channel with finite width *w*_*y*_ to the one in an unconstrained channel. One finds that ${r}_{{\Omega }_{{{{{{{{\rm{SO}}}}}}}}}}$ is essentially independent of the channel width. This result, which applies for SO fields of both BIA and SIA symmetries, can be qualitatively understood by taking into account that the spin precession rate Ω_SO_(*w*_*y*_) along the SAW propagation direction (*x*) is determined by the carrier velocity component along *x*, which remains equal to the SAW velocity as the carriers diffuse in the lateral direction (see Supplementary Information Section SM7).

We now briefly address a mechanism that can account for the weak dependence of Ω_SO_ on SAW amplitude observed for the QW. This mechanism is based on fluctuations in the storage phase *ϕ*_SAW_ of the carriers in the SAW potential during the transport. The driving force for the acoustic transport is the longitudinal component of the SAW piezoelectric field given by *F*_*x*_ = − *e*∂Φ_SAW_/∂*x*. In the previous sections, we have assumed that the electron spins remain at the SAW phase ${\bar{\phi }}_{{{{{{{{\rm{SAW}}}}}}}}}=0$ corresponding to the minimum of the piezoelectric energy − *e*Φ_SAW_, as illustrated in Fig. 5a. At these phases, however, *F*_*x*_ vanishes. To sustain a steady-state motion at the SAW velocity, both electrons (superscript *e*) and holes (*h*) must concentrate around a phase ${\bar{\phi }}_{{{{{{{{\rm{SAW}}}}}}}}}^{(i)}$ (*i* = *e*, *h*) of the SAW potential satisfying $\sin ({\bar{\phi }}_{{{{{{{{\rm{SAW}}}}}}}}}^{(i)})={r}_{d}^{(i)}={v}_{{{{{{{{\rm{SAW}}}}}}}}}/({\mu }^{(i)}{F}_{z})$ [rather than at *ϕ*_SAW_ = 0, cf. Fig. 5a]. At these phases, the SAW-induced SO fields are reduced by a factor $\cos ({\bar{\phi }}_{{{{{{{{\rm{SAW}}}}}}}}}^{(i)})=\sqrt{1-{({r}_{d}^{(i)})}^{2}}$ with respect to their maximum at *ϕ*_SAW_ = 0. As a result, the effective spin precession frequency reduces and becomes dependent on the effective carrier mobility. Due to the narrower thickness *w*_*z*_, the transport mobility μ^(*i*)^ in the QWs is expected to be lower than in the QWR and DQD cases (the ambipolar mobility scales with ${w}_{z}^{-6}$ in undoped QWs^39^), thus reducing the SAW-induced SO fields relative to the Dresselhaus contribution. According to this mechanism, the lower efficiency of the spin gates in the QW arises from the lower ambipolar mobility relative to the QWR and the DQDs due to its smaller thickness. As mentioned in connection with Fig. 4a, *ℓ*_*s*_ increases with SAW power for the spins in the QW, thus indirectly indicating a connection between the mobility and the spin dynamics. Further studies are, however, required to quantify the impact of this effect.

## Discussion

In this work, we have demonstrated the ability to acoustically transport and, simultaneously, control the polarization of electrons spins stored within moving potential dots defined by SAWs in GaAs QWs and QWRs. Within the flying control gates, the spins precess around a SAW-induced SO field, and the precession rate can be dynamically changed by a factor of over 2.5 by varying the SAW amplitude, which enabled the controlled flip of the spin polarization. The experimental results for the precession rates markedly exceed previous results and agree well with the predictions of an analytical model for the SAW-induced spin-orbit fields as well as with microscopic tight-binding calculations. The latter of which enables a precise determination of the strain related spin-orbit parameters.

We have also addressed the mechanisms governing spin relaxation in QWRs while considering the role of the carrier mobility and of the lateral interfaces on the spin dynamics. Here, the shorter spin transport lengths measured in structurally defined QWRs as compared to electrostatically defined DQDs are attributed to spin dephasing via frequent scattering at the lateral (Al,Ga)As interfaces. The latter apparently offsets the spin lifetime enhancements expected from motional narrowing in 1D channels, in agreement with a previous report for etch-defined wires with widths in the micron range^14,38^. Details of the spin scattering mechanisms in our growth-defined QWRs are, however, presently unknown and calls for additional structural studies of the QWR interfaces as well as optimization of the growth conditions. Note, however, that while DQDs exhibit long spin transport lengths, QWRs enable a wider range of dynamic precession control due to the fact that the SAW-dependent SO fields are colinear with the intrinsic Dresselhaus contribution.

The high degree of spin control in QWRs and DQDs demonstrated here enables dynamic control by simply changing the acoustic amplitude of the carrier wave. An obvious and important advantage is that both spin transport and manipulation can be performed through this flying spin gate in a single structure (e.g., a QWR) without requiring extra components (e.g., electrostatic gates for Rashba spin control). While the purely electrostatic spin control can be implemented for short transport channels^3,4^, it is not straight-forward to devise gates configuration to achieve the same functionalities in long channels, such as those required for materials with a weak Rashba SO coupling (such as GaAs). The experiments in Fig. 3b, c directly illustrate an electro-optical polarization modulator based on spin gating, which is a photonic version of the Datta-Das spin transistor. Here, the circular polarization of the PL can be set to an arbitrary value by simply varying the SAW amplitude.

The acoustic spin control technique introduced here also applies to single spins. Proposals for information processing using flying qubits have been reported^40–42^. The flying spins can also be converted to static qubits via capture into two-level centers placed within the transport path. Such a capture process has already been demonstrated for unpolarized carriers and applied for the generation of single photons at GHz rates^15,43^. The combination with dynamic spin control would then enable the generation of single photon trains with controlled polarization.

Finally, the dynamic spin control mediated by the SAW strain field could also apply to the unipolar acoustic transport of single (or of a few) electron spins, where spins are typically transported in electrostatically gated channels defined in a two-dimensional electron gas^22^. There are, however, a few differences worth mentioning to the ambipolar transport of electrons and holes investigated here. First, the so-far achieved spin transport distances for unipolar transport (of a few microns) are much shorter than the ones demonstrated here. Large spin precession angles would then require stronger acoustic fields or, alternatively, materials with a large SO coupling (e.g., InGaAs channels, as used in the purely electrostatic and ballistic spin transistors^3,4^). Second, the ambipolar transport in undoped channels demonstrated here provides a natural and direct interface between electron spins and polarized photons. In unipolar systems, such a photonic interface requires an additional arrangement for the supply of holes, as recently reported in ref. 44. In this way, both the uni- and ambipolar acoustic transport systems can realize a spin-based quantum information processor with a photonic interface.

## Methods

### QWR sample fabrication

The planar QWRs used in this work were fabricated using MBE by overgrowing a 10 nm-thick QW on a GaAs(001) substrate pre-patterned with shallow (approximately 30 nm high) rectangular ridges [cf. Fig. 2a]^30^. The anisotropic nature of the MBE growth induces a local thickening of the QW at the ridge sidewalls oriented along the $\tilde{{{{{{{{\bf{x}}}}}}}}}|\vert [110]$-direction along the surface, thus forming a QWR parallel to this sidewall. The thickness and width of the QWR are (25 ± 5) nm and (200 ± 5) nm, respectively, as determined by scanning transmission electron microscopy^30^. Small and unintentional fluctuations in the potential in the QW plane or along the QWR act as efficient recombination sites during acoustic transport^30^. The PL from these fluctuations was used to extract the spin state information of the electron. The moving potential dots for carrier and spin transport are created by propagating a SAW with a wavelength *λ*_SAW_ = 4 μm (frequency of 726 MHz at 15 K and velocity *v*_SAW_ = 2904 m/s) along the wire axis. The SAW is generated by a split-finger interdigital transducer deposited on the sample surface. Its amplitude can be quantified in terms of the linear acoustic power density *P*_SAW_, defined as the ratio between the coupled acoustic power and the width of the SAW beam. During the acoustic transport, the carriers are confined within dots with dimensions equal to the QWR width and less than ~ *λ*_SAW_/2 along the directions perpendicular and parallel to the SAW propagation, respectively. Acoustic spin transport was also investigated in the QW embedding the QWR. In this case, carrier motion is unconstrained in the direction perpendicular to the transport.

### DQD sample fabrication

The DQDs were created via the interference of two orthogonal SAW beams propagating along the $\tilde{{{{{{{{\bf{x}}}}}}}}}|\vert [110]$ and $\tilde{{{{{{{{\bf{y}}}}}}}}}|\vert [\bar{1}10]$ directions of a 30 nm thick QW on GaAs(001)^15^. The interference of the piezoelectric fields of the SAWs creates an array of DQDs propagating along the $\tilde{{{{{{{{\bf{{x}}}}}}}^{\prime}}}}|\vert [010]$ surface direction^15^. Along the DQD transport path, a metal strip was deposited on the surface of the sample to screen the piezoelectric field and induce electron-hole recombination. DQDs yield the longest, so far, reported acoustic spin transport distances^15,45^. SAWs with a wavelength of *λ*_SAW_ = 5.6 μm were used and yield DQDs with dimensions of approximately *λ*_SAW_/2 × *λ*_SAW_/2 propagating with a velocity ${v}_{{{{{{{{\rm{DQD}}}}}}}}}=\sqrt{2}{v}_{{{{{{{{\rm{SAW}}}}}}}}}=$ 4115 m/s.

### Photoluminescence measurements

The spectroscopic photoluminescence (PL) studies of the spin transport were performed at low temperatures (10–20 K) in a microscopic PL setup with radio-frequency (rf) wiring for SAW excitation. The spins were optically excited using a circularly polarized laser beam with tunable wavelength (*λ*_*L*_ between 760 and 808 nm) focused onto a ~2 μm wide spot on the sample surface. The PL emitted along the SAW path with left- (*I*_*L*_) and right-hand (*I*_*R*_) circular polarization was collected with spatial resolution and used to determine the spin polarization^46^${\rho }_{s}=({I}_{PL}^{\circlearrowleft }-{I}_{PL}^{\circlearrowright })/({I}_{PL}^{\circlearrowleft }+{I}_{PL}^{\circlearrowright })=({I}_{R}-{I}_{L})/({I}_{R}+{I}_{L})$(see Supplementary Information Section SM1). As mentioned in the main text, the spin polarization in QWRs was determined by collecting the PL from trap centers along the transport path. Such traps are not available in the high-quality QW transport path for DQDs. In this case, a metal strip was added to the transport path to screen the SAW piezoelectric field and, in this way, blocks the transport and induces carrier recombination. We found that the blocking action of the metal strip become less effective at high acoustic amplitudes, thus limiting the range of SAW power in Fig. 4. In addition, the accumulation of electrons and holes near the strip induces electron spin dephasing via D’yakonov-Perel’ as well as hole spin scattering before recombination, thereby reducing the spin polarization.

### Tight-binding calculations

The analytical model for the SO fields applies for a QW with infinite potential barriers and, thus, neglects the effects of the QW interfaces. In addition, the model neglects the impact of the SAW field on the electronic states, which can be significant for the valence band states in wide QWs^47^. The the SO field Ω_SO_ contributions were calculated in QWs using the tight-binding (TB) method^35,36^. The effects of the SAW were taken into account by using the strain and piezoelectric fields to determine the atomic positions and on-site potentials, respectively, within the TB supercell (see Supplementary Information Section SM5).

### Monte Carlo spin dynamics

In the calculations, the spins are assumed to move along the *x*-direction with the SAW velocity while having a random motion along the *y*-direction with mean-free-path *ℓ*_*p*_ and thermal velocity *v*_*p*_ defined by the electron mobility μ and temperature (see Supplementary Information Section SM7). Figure 6a, b display the simulated dependence of *ℓ*_*s*_ and Ω_SO_ for transport in channels with different widths *w*_*y*_ under BIA and SIA SO fields. The SO fields were assumed to have an amplitude ${{\Omega }_{{{{{{{{\rm{SO}}}}}}}}}}^{(2{{{{{{{\rm{D}}}}}}}})}=2\pi {v}_{{{{{{{{\rm{SAW}}}}}}}}}/{L}_{{{{{{{{\rm{SO}}}}}}}}}^{(2{{{{{{{\rm{D}}}}}}}})}$ dictated by a spin precession period ${L}_{{{{{{{{\rm{SO}}}}}}}}}^{(2D)}=12\,\mu$m similar to the ones measured for QWs in Fig. 3. We further assumed μ = 4 m^2^/(Vs) and *T* = 4 K, which yield *ℓ*_*p*_ = 0.1 μm.

## Supplementary information

Supplementary Information

## Data Availability

The numerical simulation and measurement data that support the finding within this study are included within the manuscript and Supplementary Information and can also be made available upon reasonable request from the corresponding authors.

## Code availability

The code used for the numerical modeling within this study are available upon reasonable request from the corresponding authors.
