# Supplementary material for: Flying electron spin control gates
Source: Nat Commun. 2022 Sep 14;13:5384. doi: 10.1038/s41467-022-32807-x (PMC9475040; doi:10.1038/s41467-022-32807-x)
Supplement: Supplementary file 1 — Supplementary Information [file 41467_2022_32807_MOESM1_ESM.pdf]

Supplementary Material: Flying electron spin control gates

Paul L. J. Helgers,[1,2] James A. H. Stotz,[3,4] Haruki Sanada,[2]
Yoji Kunihashi,[2] Klaus Biermann,[3] and Paulo V. Santos[3,*]

[1] *Paul-Drude-Institut für Festkörperelektronik, Leibniz-Institut im Forschungsverbund Berlin e.V., Hausvogteiplatz 5–7, 10117 Berlin, Germany*
[2] *NTT Basic Research Laboratories, NTT Corporation, 3–1 Morinosato-Wakamiya, Atsugi, Kanagawa 243-0198, Japan*
[3] *Paul-Drude-Institut für Festkörperelektronik, Leibniz-Institut im Forschungsverbund Berlin e. V., Hausvogteiplatz 5–7, 10117 Berlin, Germany*
[4] *Department of Physics, Engineering Physics & Astronomy, Queen's University, Kingston, ON, K7L 3N6 Canada*

(Dated: May 6, 2022)

SM1. SPIN POLARIZATION MEASUREMENTS

The spectroscopic photoluminescence (PL) studies of the spin transport were carried out in a helium flow cryostat (10-20 K) with optical access and radio-frequency wiring for SAW excitation. The spins were optically excited using a circularly polarized laser beam from a tunable Ti-sapphire laser (wavelengths λ_L between 760 and 808 nm) focused onto a $\sim$ 2 μm wide spot on the sample surface by a 20x objective [L in Fig. 2(c) of the main text]. The same objective collects the PL emitted from along the SAW path and directs it to a triple-grating spectrometer operating in the subtractive mode and equipped with a cooled CCD detector. Spatially resolved PL maps are recorded by imaging the acoustic transport path onto the input slit of the spectrometer. An optical arrangement consisting of a quarter wave plate and a beam polarization displacer placed in front of the spectrometer slit shifts the PL images with right (I_R) and left (I_L) hand circular polarizations along the slit direction. In this way, we simultaneously record PL images of the spatial distribution of the two polarizations on the CCD.

The spin polarization ρ_s during acoustic transport can be determined by exciting spins with a right hand circularly polarized laser beam and detecting the PL intensities I_R and I_L according to:

$$\rho_s = \frac{I_R - I_L}{I_R + I_L}. \tag{SM1}$$

Since hole-spin relaxation in QWs and QWRs is typically much faster than for electrons [1], we assume that ρ_s reflects only the electron spin dynamics.

In order to correct for artifacts arising from dichroism of the optical components of the setup, the polarization experiments were carried out by recording PL maps excited with a right-hand and then a left-hand circularly polarized laser beam. We will denote the corresponding PL intensities as I_{LL}, I_{LR}, I_{RL} and I_{RR}, where the first (second) index corresponds to the circular polarization of the excitation (PL emission). For an ideal optical system (i.e., with transmission independent of the polarization), one expects a ratio

$$a_r^2 = \frac{I_{LL} I_{RL}}{I_{RR} I_{LR}} = \frac{t_L}{t_R} \tag{SM2}$$

equal to unity. The presence of dichroic elements in the optical path makes $a_r \neq 1$. The effect of these components on the spin polarization can be corrected by using the following expression to determine the spin polarization:

$$\rho_s = \frac{a_r I_{RR} - I_{RL}}{a_r I_{RR} + I_{RL}}. \tag{SM3}$$

SM2. SPIN POLARIZATION IN QWRS

Figure SM1 displays the dependence of the spin orientation during acoustic transport propelled by SAWs with different acoustic amplitudes. The measurement conditions are identical to the ones described in the main text for the acquisition of the data for Fig. 3b. As in this figure, ρ_s oscillates with transport distance x with a period that reduces with increasing SAW amplitudes, thus demonstrating the dynamic control of the spin vector. Note again that for the maximum acoustic amplitude one reaches a 180 deg spin rotation for a transport distance of less than 10 μm. Note that while the precession period changes with the acoustic amplitude, the spin transport lengths ℓ_s remain essentially unaffected, as displayed in Fig. 4a of the main text.

* santos@pdi-berlin.de

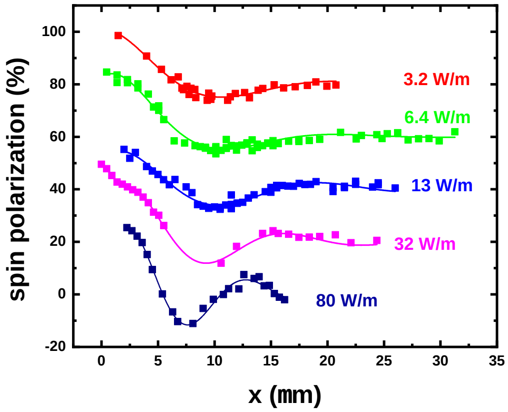

FIG. SM1. Profiles of spin polarization transport in a QWR measured for different linear acoustic powers (in units of W/m).

SM3. SPIN LIFETIMES

The spin lifetimes τ_s in the absence of acoustic excitation were determined by time-resolved Kerr rotation measurements carried out in the pump-probe configuration [2]. Typical temporal Kerr traces for the QW (black) and QWR (red) are shown in Fig. SM2. The measurements were performed using an excitation energy of 1.551 eV and power density of $8\,\mathrm{W\,cm^{-2}}$. The traces can be fitted with an exponential decaying function yielding Kerr rotation decay times $\tau_K = 200$ ps for the QW and $\tau_K = 1$ ns for the QWRs.

The spin decay time was determined by combining the Kerr rotation decay times with the carrier recombination lifetimes obtained from time-resolved PL measurements.

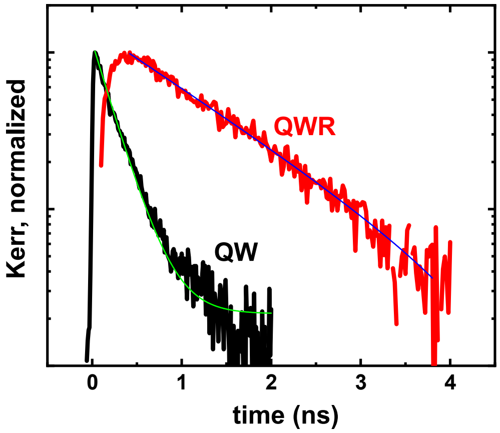

FIG. SM2. Time-resolved Kerr polarization rotation measured on the QW (black) and QWR (red). The lines are exponentially decaying functions yielding a Kerr rotation decay time of approximately 200 ps and 1 ns for the QW and the QWR, respectively.

This procedure yields spin lifetimes of $\tau_s = 0.5$ ns and $\tau_s = 2$ ns for the QW and QWRs, respectively. The much longer spin lifetime in the QWR as compared to the QW is attributed to (i) the lateral confinement of the QWR carriers by the surrounding QW, leading to motional narrowing and (ii) the larger thickness of the QWR. Whereas both effects suppress D'yakonov-Perel' spin relaxation [3, 4], the latter also decreases of the electron-hole exchange interaction and its associated spin relaxation ratio [5].

SM4. SAW-INDUCED SO FIELDS

In this section, we briefly summarize the SO mechanisms related to the strain and piezoelectric fields induced by a SAW. In the absence of a SAW, the conduction band spin splitting for electrons moving along the $\langle 110 \rangle$-directions of an intrinsic GaAs QW on a (001) substrate is given by the Dresselhaus term:

$$\Omega_\mathrm{D} \sim \pm\gamma k \left[\left(\frac{\pi}{w_z} \right)^2 - \frac{1}{2} k^2 \right], \qquad \text{(SM4)}$$

where γ is a material constant and $w_z = d_{QW} + 2d_0$ is the total extension of the electronic wave function, assumed to be equal to the QW width (d_{QW}) plus the penetration depth in the barrier layers (d_0). For an electron moving with the SAW velocity v_SAW, the wave vector k is obtained from the electron momentum according to:

$$k = \frac{m^* v_\mathrm{SAW}}{\hbar}. \qquad \text{(SM5)}$$

where m^* is the conduction band (CB) effective mass.

A uniaxial strain leads to a linear term in the CB splitting given by:[6–8]

$$H_\mathrm{S} = \frac{1}{2} \left[C_3 (\hat{\sigma}\vec{\phi}) + C_3' (\hat{\sigma}\vec{\psi}) \right] \qquad \text{(SM6)}$$

where $\phi_z = \varepsilon'_{xz} k_z - \varepsilon'_{yz} k'_y$ and $\psi_z = k_z(\varepsilon'_{xx} - \varepsilon'_{yy})$ and cyclic permutations. The $\hat{\sigma}_i$ denote the Pauli matrices. The apostrophe ($'$) indicates that the strain components are relative to the conventional axes x', y', and z'. This Hamiltonian gives rise to a strain-induced spin precession frequencies given by [9]

$$\hbar\Omega_\mathrm{S} = C_3 \begin{bmatrix} \varepsilon'_{yx} k_y - \varepsilon'_{zx} k_z \\ \varepsilon'_{zy} k_z - \varepsilon'_{yx} k_x \\ \varepsilon'_{xz} k_x - \varepsilon'_{yz} k_y \end{bmatrix} + C_3' \begin{bmatrix} k_x(\varepsilon'_{yy} - \varepsilon'_{zz}) \\ k_y(\varepsilon'_{zz} - \varepsilon'_{xx}) \\ k_z(\varepsilon'_{xx} - \varepsilon'_{yy}) \end{bmatrix} \qquad \text{(SM7)}$$

In order to address the SAW strain field, it is convenient to use a reference frame with the x-axis along the SAW propagation direction, the y-axis on the sample surface and perpendicular to x, and the z-axis perpendicular

to the surface. A SAW propagating along the [110]- or [$\bar{1}$10]-directions of the (001) surface induces three non-vanishing engineering strain components u_{xx}, u_{zz}, and u_{xz}. Furthermore, we use u_{ij} to denote the engineering strain components and, thus, to distinguish them from the physical strain ones $\varepsilon_{i,j}$ ($\varepsilon_{ij} = u_{ij}$ for $i = k$ and $\varepsilon_{ij} = u_{ij}/2$ for $i \neq j$).

In a piezoelectric material, the strain field from the SAW generates a piezoelectric polarization field D given by

$$D = e_{14}[u_{xz}, 0, u_{xx}]^T, \tag{SM8}$$

where the superscript T denotes the vector transposition operation. When the SAW fields are transformed to the Cartesian reference frame, the strain and electrical polarization fields become:

$$\varepsilon' = \left[\frac{u_{xx}}{2}, \frac{u_{xx}}{2}, u_{zz}, \pm\frac{u_{xz}}{\sqrt{2}}, \frac{u_{xz}}{\sqrt{2}}, \pm u_{xx}\right]^T, \tag{SM9}$$
$$D' = e_{14}[2u_{xz}, 0, u_{xx}]^T, \tag{SM10}$$

where upper and lower signs apply for SAWs propagating along the [110]- and [$\bar{1}$10]-directions, respectively. By substituting Eq. SM9 into Eq. SM7, one obtains:

$$\hbar\Omega_\mathrm{S} = \frac{1}{2\sqrt{2}}k\left[C_3u_{xx} + C_3'(u_{xx} - 2u_{zz})\right]\begin{bmatrix}1\\-1\\0\end{bmatrix} \tag{SM11}$$

Note that the strain induces a SO component perpendicular to the SAW propagation direction.

A SAW in a piezoelectric material also induces a transverse piezoelectric field F_z associated with D, which gives rise to a Rashba spin-orbit contribution. As a consequence, SAWs along a $\langle 110\rangle$ induce three contributions for the CB spin splitting, all oriented in-plane and perpendicular to the SAW propagation direction, given by:

$$\hbar\Omega_\mathrm{SO} = \Omega_\mathrm{D} + \Omega_\mathrm{S} + \Omega_\mathrm{R} \tag{SM12}$$
$$\Omega_\mathrm{S} = \mp k\frac{1}{2}\left[C_3u_{xx} + C_3'(u_{xx} - 2u_{zz})\right] \tag{SM13}$$
$$\Omega_\mathrm{R} = -2kr_{41}F_z \tag{SM14}$$

The upper and lower signs apply for SAWs along the [110] and [$\bar{1}$10] directions, respectively. We will show in Sec. SM5 A that the C_3' term in Eq. SM13 is much smaller than the one associated with C_3. This term will be neglected in the main text and in the subsequent analyses.

TABLE SM1. Tight-binding parameters used in the calculation of spin-orbit conduction band splitting. The notation used corresponds to the one of Ref. 10. In order to account for the valence band discontinuity we subtracted 0.007 eV from the diagonal parameters (i.e., E_s, E_p, and E_{s^*}) of the $Al_{0.15}Ga_{0.85}As$ barrier layers.

Parameter	GaAs[11] (eV)	$Al_{0.15}Ga_{0.85}As$ (eV)
E_s(anion)	-8.457	-8.3172
E_p (anion)	0.9275	0.92938
E_s(cation)	-2.7788	-2.53673
E_p (cation)	3.5547	3.55250
V_ss	-6.4513	-6.48321
V_xx	1.9546	1.95121
V_xy	4.77	4.69110
$V_{\mathrm{s_0p}}$(anion,cation)	4.48	4.57360
$V_{\mathrm{s_1p}}$(cation,anion)	7.85	7.49390
E_{s^*}(anion)	8.4775	8.32833
$V_{\mathrm{s_0^*p}}$(anion,cation)	4.8422	4.78967
$E_{\mathrm{s_1^*}}$(cation)	6.6247	6.64005
$V_{\mathrm{s^*p}}$(cation,anion)	7	6.69940
$3\lambda_a$ (anion)	0.39	0.39171
$3\lambda_c$ (cation)	0.174	0.15150

SM5. TIGHT-BINDING CALCULATIONS OF SPIN SPLITTINGS IN QWS

We present in this section details about the calculations of the spin splitting of the conduction band of GaAs QWs under SAW fields. In bulk GaAs, this splitting results from a three-band coupling process involving the s-like GaAs conduction band and the p-like conduction and valence bands [12]. The three-band interaction process leads to the cubic dependence of the spin splitting on wave vector k. Calculation of the splitting requires, therefore, that one takes into account interactions between of all these bands. As demonstrated in Ref. 12, this can be carried out using ab-initio approaches or empirical approaches using a large number of bands (e.g., a 14 band $\mathbf{k}\cdot\mathbf{p}$ method). Here, we determine the splittings using the empirical tight-binding (TB) method[13] following the approach described in Refs. 14 and 15. This atomistic approach enables the determination of the band structure of QWs with widths up to a few tens of nanometres using moderate computational efforts. Although empirical in the sense that it uses parameters fitted to the bulk band structure, it can be regarded as "microscopic" when compared with other approaches such as the $\mathbf{k}\cdot\mathbf{p}$ effective mass calculations.

The TB calculations were carried out using a basis consisting of sp^3s^* orbitals [16] including spin-orbit coupling[17, 18]. This orbital basis consists of 10 orbitals per atom and includes only nearest-neighbour interactions. It has been shown to reproduce very well the highest valence and the lowest conduction bands of most bulk semiconductors[16]. The tight-binding parameters employed for the GaAs QW and the $Al_{0.15}Ga_{0.85}As$ barrier layers are summarized in Table SM1.

The calculations were performed for an electron with

wave vector given by Eq. SM5 in a periodic supercell consisting of a single QW with sandwiched between 4.5 nm wide $Al_{0.15}Ga_{0.85}As$ barriers. The atomic positions within the unit cell were deformed in order to take into account the effects of the SAW strain field determined in the previous section. The effects of the piezoelectric field F_z were incorporated in the calculations by adding the position-dependent electrostatic energy to the on-site TB parameters. The following convention applies for the crystallographic orientation of the axes:

- in the unit cell, the anion (As) sits at the origin while the cation (Ga) is at $(a_0/4)(111)$ (this is the same as in Ref. 12);
- a positive (piezo) electric field corresponds to a field along the growth direction.

A. Tight-binding calculation of the spin-orbit strain coefficients

As discussed in Sec. SM4, the SAW strain induces SO contributions described by two material constants C_3 and C_3'. Reported values for these contributions span a wide range: $C_3 = 0.47$ eVnm [19], $C_3 = 0.52$ eVnm [6], and $C_3 = 0.81$ eVnm [9]. C_3' has not been measured (nor calculated), and should be negligible in comparison with C_3.In this section, we use the TB approach to estimate the magnitude of these contributions.

We determined the strain coefficients C_3 and C_3' by performing TB calculations for GaAs under strain and fitting to the expressions in Eq. SM12. C_3' was calculated using a strain field with $u_{xx} = 0$, $u_{xz} = 0$, and for different values for u_{zz}. The results are summarized in Fig. SM3(b). We find that C_3' is essentially zero.

In order to calculate C_3, we took $u_{zz} = 0$, $u_{xz} = 0$ and performed calculations for different u_{xx} [Fig. SM3(a)]. The TB calculations yield $|C_3| = 1.65$ eVnm. This C_3 value agrees very well with the experimental one obtained from the QWR data in the main text. It is, however, twice as large as the one experimentally determined by Beck (0.81 eVnm), [9] and also larger then the one estimated from D'yakonov's calculations. [7, 20] Corresponding results for QW structures are presented in the main text.

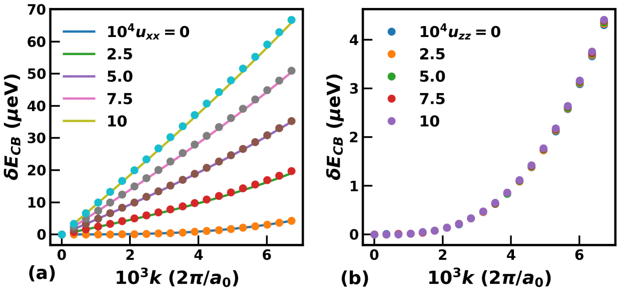

FIG. SM3. Calculations of the conduction band spin splittings in GaAs under different strain components (a) u_{xx} and (b) u_{zz} (symbols). The lines are fits to Eq. SM13 (Dresselhaus and strain parts) used to determine the coefficients $|C_3| = 1.65$ eVnm and $C_3' = 0$.

SM6. EXPERIMENTAL DETERMINATION OF STRAIN-INDUCED C_3 PARAMETER

A. QWs and QWRs data

According to Eq. SM12 (and to Eq. 2 of the main text), the SO field for spins under the moving SAW field can be stated in the reference frame with axis $x = [110]$, $y = [\bar{1}10]$ and $z = [001]$ as:

$$\hbar\Omega_{SO} = \left[\gamma \left[\frac{\pi}{w_z}\right]^2 - 2F_z r_{41} - \frac{C_3 u_{xx}}{2}\right] k_x. \quad \text{(SM15)}$$

Here, k_x is given by Eq. SM5 and $w_z = d_{QW} + 2d_B$ is the extension of the electron wave function along the z-direction given by the sum of the nominal QW width (d_{QW}) and the penetration depths in the upper and lower QW barriers (d_B). In the calculations that follow, we assume $d_B = 2$ nm. The spin-orbit parameters are given by $\gamma = 17 \times 10^{-30}\,\mathrm{eV\,m^3}$ (Dresselhaus [21]), r_{41} = -$5.95 \times 10^{-20}\,e\,\mathrm{m^2}$ (Bychkov-Rashba [4]) and C_3 the strain parameter.

As mentioned in the main text, F_z is proportional to u_{xx}. In order to determine the proportionality constant, we conducted numerical simulations of the acoustic and piezoelectric fields of a SAW with a wavelength $\lambda_{\mathrm{SAW}} = 4\ \mu$m propagating along the [110] -direction (see Ref. 22 for details). Figure SM4(a) shows the relative values of the SAW potential Φ_{SAW} (black), u_{xx} (blue), and F_z (red) at various positions/phases of the wave. The curves are normalized to their maximum values and shifted along the vertical axis for clarity. For this propagation direction, the maxima of Φ_{SAW}, corresponding to the transport phase of electrons (brown circles with minus sign), F_z is negative (i.e., oriented along the $-z$ direction). The uniaxial strain u_{xx} is positive at this position. Thus, $u_{xx} > 0$ and $F_z < 0$ in Eq. (SM15).

Figure SM4(b) shows the amplitude of the piezoelectric field $|F_z|$ as a function of the strain u_{xx}. The up-

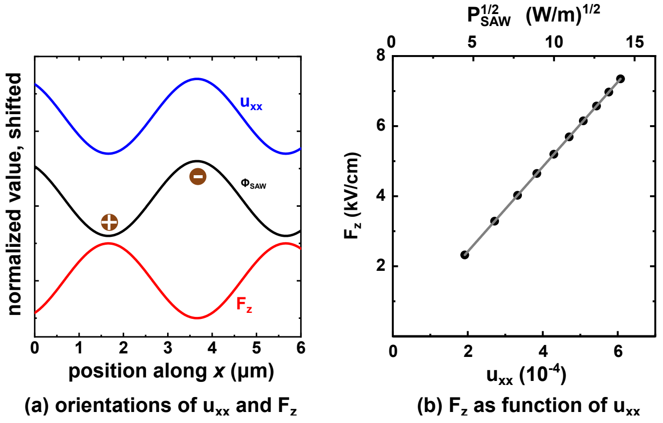

FIG. SM4. (a) Relative orientations of Φ_{SAW} (black), F_z (red) and u_{xx} (blue) along the QWR axis. The transport position of electrons (holes) in the case of ideal acoustic transport is depicted by brown circles with a minus (plus) sign. (b) Dependence of $|F_z|$ on u_{xx}. The upper horizontal scales displays the square root $\sqrt{P_\ell} \propto u_{xx,0}$, thus establishing the relationship between the strain amplitude and the linear acoustic power density generated by the IDT.

per horizontal scale displays the corresponding values for $\sqrt{P_\ell} \propto u_{xx}$, thus establishing the relationship between the strain amplitude and the linear acoustic power density P_ℓ excited by the IDT. The linear fit yields a slope of $r_{\rm SF} = 12 \times 10^3\,{\rm kV\,cm^{-1}}$. Note that the strain is small, so that the resulting field is on the order of kV cm^{-1}. Substitution of the simulation results modifies Eq. (SM15) in:

$$\hbar\Omega_{SO} = \left[\gamma\left[\frac{\pi}{w_z}\right]^2 + 2r_{\rm SF}u_{xx}r_{41} - \frac{C_3 u_{xx}}{2}\right]k_x. \quad \text{(SM16)}$$

Note that the second term on the right-hand-side (corresponding to Bychkov-Rashba) turns positive due to the negative F_z.

The fits of the experimental data for QWRs in Fig. 4(b) of the main text to Eq. (SM4) were carried out using an effective electron mass $m_e^* = 0.067$ and a SAW velocity $v_{SAW} = 2904\,{\rm m\,s^{-1}}$ yielding $C_3 = -2.6\,{\rm eV\,nm}$. Table SM2 summarizes the calculated values for the different components of the SO field for two different SAW powers. All SO contributions are directed along the same direction (the $+y$-axis). In addition, the strain-related SO contribution is much larger than the one from the piezoelectric field.

B. Calculation of C_3 parameter for DQDs

Due to the different orientations of the Dresselhaus and the SAW-related SO fields expressed in Eq. 3 of the main text, the amplitude of the precession frequency experienced by spins in DQDs created by the superposition of SAW beams with the same intensity propagating along the x- and y-directions becomes:

QW/QWR	Ω_D (GHz)	Ω_R (GHz)	Ω_S (GHz)	Ω_{exp} (GHz)	Ω_{calc} (GHz)
QWR, $P_{\rm SAW}$ = 7 dBm	0.51	0.03	0.20	0.89	0.74
QWR, $P_{\rm SAW}$ = 21 dBm	0.51	0.14	1.03	1.77	1.68
QW, $P_{\rm SAW}$ = 7 dBm	2.20	0.03	0.20	1.66	2.43
QW, $P_{\rm SAW}$ = 21 dBm	2.20	0.14	1.03	1.71	3.38

TABLE SM2. Spin-orbit precession frequencies in QWs and QWRs for different SAW powers.

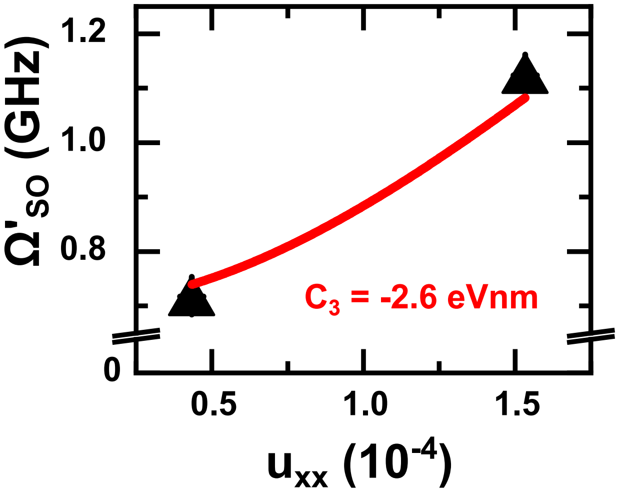

FIG. SM5. Fit of the DQD data to determine the C_3 parameter.

$$|\Omega'_{SO}| = \sqrt{2\Omega_D^2 + 4\left[\Omega_R + \Omega_S\right]^2}, \quad \text{(SM17)}$$

where, as mentioned in the main paper, $\Omega_{\rm D}$, $\Omega_{\rm R}$, and $\Omega_{\rm S}$ are the magnitudes of the precession frequency for a single SAW beam along the x-direction. As in the previous case, the amplitude of the transverse piezoelectric field can be related to the strain amplitude u_{xx} by a factor $r_{\rm SF} = 21 \times 10^3$ kV/cm. Due to the quadratic dependence of Ω'_{SO} on $\Omega_{\rm D}$ in Eq. (SM17), it is not possible to determine from the DQD data in Fig. 4(b) of the main paper the relative signs of the Dresselhaus and SAW-related SO fields. The red line in Fig. SM5 shows, however, that the SAW power dependence of Ω'_{SO} is reasonably well accounted for using a value of $C_3 = -2.6\,{\rm eV\,nm}$ that is comprable to the QWR results. Table SM3 summarizes the values of the different SO-precession frequencies for DQDs.

DQDs	Ω'_D (GHz)	Ω'_R (GHz)	Ω'_S (GHz)	Ω'_{exp} (GHz)	Ω'_{calc} (GHz)
$P_{\rm SAW}$ = 2 W/m ($C_3 = -2.0$)	0.53	-0.05	-0.23	0.71	0.60
$P_{\rm SAW}$ = 25 W/m ($C_3 = -2.0$)	0.53	-0.20	-0.79	1.11	1.12

TABLE SM3. Spin-orbit precession frequencies determined for transport by DQDs under different SAW powers.

SM7. MONTE-CARLO SIMULATIONS OF THE ACOUSTIC SPIN TRANSPORT

We study, in this section, the spin dynamics during acoustic transport in narrow channels using Monte-Carlo simulations of the spin trajectories. Similar Monte-Carlo investigations of the confinement effects on the spin dynamics were carried out for diffusing spins by Kiselev and Kim [3, 23]. In contrast to the results presented here, those previous studies were restricted to the transport dynamics for short spin coherence times, where spatial precession oscillations are not observed. In addition, these studies did not address the impact of carrier drift under an external field on the spin dynamics.

The Monte-Carlo simulations were performed by assuming that $z||[001]$-oriented electron spins are optically excited at the position $(x, y) = (0, 0)$ at the center of a channel with width w_y. A surface acoustic wave (SAW) propagating with phase velocity $v_{\rm SAW}$ along $x||[110]$ collects the spins and transports them along the x-direction with the SAW velocity. During transport, the electron spins precess around the SO-field with angular precession frequency $\Omega_{\rm SO}$ given by:

$$\Omega_{\rm SO} = (1 - |r_s|)\Omega_{\rm BIA} + r_s\Omega_{\rm SIA}, \qquad \text{(SM18)}$$

where $\Omega_{\rm BIA}$ and $\Omega_{\rm SIA}$ are the components of the SO-field due to the bulk (BIA) and structural (SIA) inversion asymmetry, respectively, and r_s is a factor weighting the amplitude of the two components (see further details below). For small magnitudes of the electron momentum $\hbar\mathbf{k}$, these components can be expressed in the $\mathbf{k} = (k_x, k_y) = k(\cos\theta, \sin\theta)$ basis, where θ is the propagation angle with respect to the x axis, as:

$$\Omega_{\rm BIA} = \underbrace{c_{\rm BIA}k}_{\Omega^{(\rm max)}_{\rm BIA}} \begin{bmatrix} +\sin(\theta) \\ +\cos(\theta) \end{bmatrix} \qquad \text{(SM19)}$$

$$\Omega_{\rm SIA} = \underbrace{c_{\rm SIA}k}_{\Omega^{(\rm max)}_{\rm SIA}} \begin{bmatrix} -\sin(\theta) \\ +\cos(\theta) \end{bmatrix} \qquad \text{(SM20)}$$

Here, $c_{\rm i}$ ($i = {\rm BIA, SIA}$) are material constants associated with the BIA and SIA components determining the precession frequencies $\Omega^{(\rm max)}_{\rm i} = c_i k$ (i.e., only linear terms in k are taken into account).

Due to the dependence on carrier momentum, the determination of the spin dynamics under the SO fields requires the knowledge of the electron trajectory during the motion. The latter was determined based on the following assumptions:

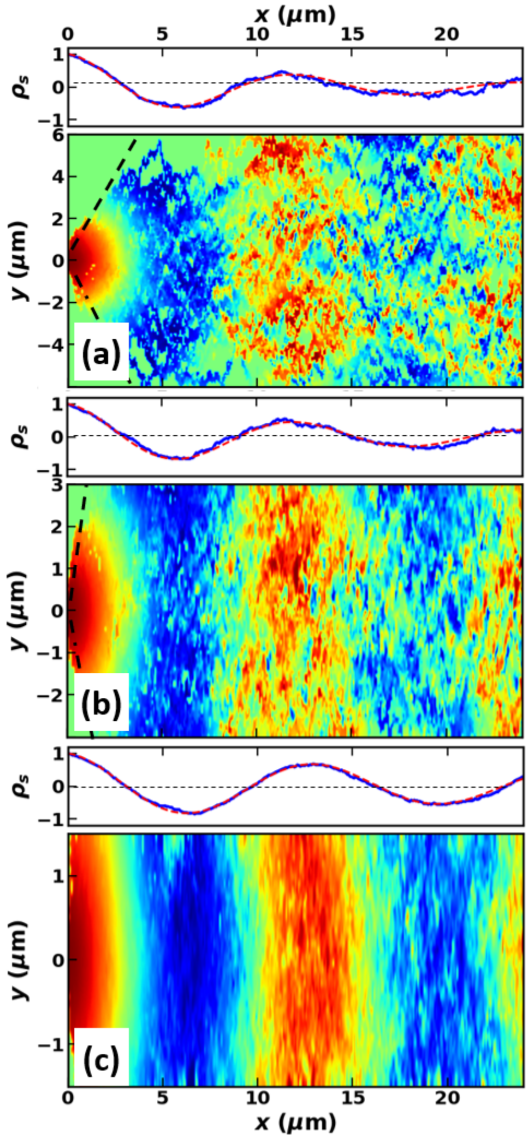

FIG. SM6. Spin polarization profiles during acoustic transport in channels with width (a) $w_y = L^{(2D)}_{SO} = 12$ μm, (b) $w_y = 6$ μm, and (c) $w_y = 3$ μm. The profiles result from Monte-Carlo simulations of the spin transport under a spin-orbit field with SIA asymmetry quantified by an effective spin-orbit length $L_{\rm SO} = 12$ μm. The simulations assume a temperature of 10 K and carrier mobility of 4 $m^2/(Vs)$. The colors encode the spin projection along z. The upper plots display the spin polarization ρ_s obtained by integrating the color maps within the range $|y| < 2$ μm.

- the propagation velocity $v_{\rm SAW}$ along x imparts a momentum along this direction given by $\hbar k_x = m^*_e v_{\rm SAW}$, where m^*_e is the electron effective mass;

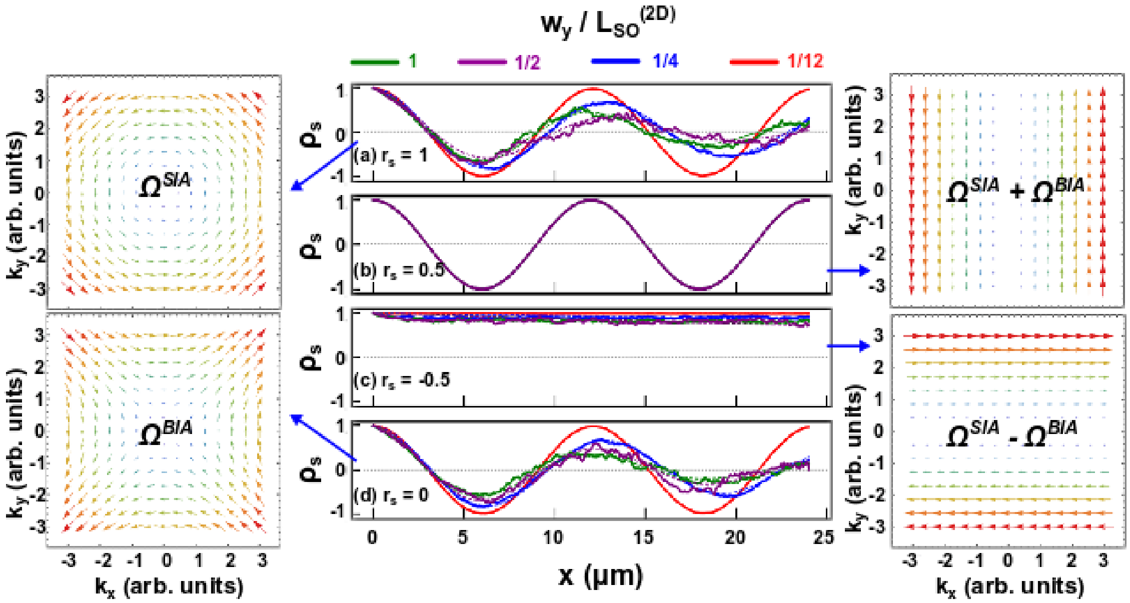

FIG. SM7. Spin polarization profiles along the SAW transport path calculated for different ratios w_y/L_{SO} and (a) $r_s = 1$ $[\Omega_{\rm SO} = \Omega_{\rm SIA}$, cf. Eq. (SM20)] (b) $r_s = 0.5$ $(\Omega_{\rm SO} = (\Omega_{\rm SIA} + \Omega_{\rm SIA})/2)$, (c) $r_s = -0.5$ $(\Omega_{\rm SO} = (\Omega_{\rm SIA} - \Omega_{\rm SIA})/2)$, and (d) $r_s = 0$ $[\Omega_{\rm SO} = \Omega_{\rm BIA}$, cf. Eq. (SM19)]. (b) and (c) are the spin helix modes with $\mathbf{\Omega}_{\rm SO}$ oriented along the y- and x-directions, respectively. The right and left inset display the corresponding momentum dependence of the spin-orbit field for these four configurations.

- the spins are free to diffuse along $y||[\bar{1}10]$ (i.e., along the SAW wave fronts) with a velocity v_y (and corresponding momentum $m_e^* v_y$) determined from a thermal distribution given by:

$$f(v_y)\delta v_y = \left[\frac{m_e^*}{2\pi k_B T}\right]^{1/2} e^{-\frac{m_e^* v_y^2}{2k_B T}} = \frac{1}{\sqrt{\pi} v_p} e^{-v^2/v_p^2} \delta v_y, \tag{SM21}$$

  where $v_p = \sqrt{\frac{2k_B T}{m_e^*}}$ and T is the temperature. The diffusion along y proceeds until the spins are back-reflected at the edges of the channel.

- within the channel boundaries, the electron spins precess around the spin orbit field with an angular precession frequency $\Omega_{\rm SO}$ [cf. Eq. (SM18)] determined by the momentum $\hbar\mathbf{k}$ until they are scattered with a scattering time τ. The scattering time τ is determined from the mobility μ according to: $\tau = m_e^* \mu / q_e$, where q_e is the electron charge. The motion along y is randomized at each scattering event.

- Due to the linear dependence of the precession frequencies on $\mathbf{k}$, the spin precession angle for propagation along a fixed direction only depends on the distance between the initial and final points of the trajectory. If $(\cos\theta, \sin\theta)\Delta\ell$ is the displacement vector between two successive scattering events, than the corresponding spin precession angle under a SO field $\Omega_{\rm SIA}$ will be $\delta\phi_s = 2\pi\Delta\ell/L_{SO}^{\rm SIA}$. Here, $L_{SO}^{\rm SIA} = m_e^* c^{\rm SIA}/\hbar$ is the SO length, which only depends on material properties. These results apply for $\Delta\ell << L_{\rm LO}^{SIA}$ and are also valid for the BIA component. The spin dynamics results to be shown below were calculated assuming $L_{\rm SO}^{SIA} = L_{\rm LO}^{BIA} = L_{\rm SO}^{(2D)} = 12$ μm.

Figures SM6(a)-(c) compare spin polarization maps calculated for acoustic transport under a SO field with SIA symmetry (Ω_{SIA}) along channels with different widths w_y. In each map, the colors encode the z projection of the spin vector calculated by averaging over 40 Monte-Carlo realizations. In the wide channels [Figs. SM6(a)], the individual spin trajectories can still be identified. Here, one sees that the spins initially propagate radially from the excitation spot $(x, y) = (0, 0)$ within a cone (dashed lines) defined by the ratio between the average thermal velocity and the SAW velocity. The spin polarization within this cone oscillates along the radial direction with a precession period given by L_{SO}. For longer transport distance along x, a increasing number of trajectories with different spin polarization superimpose, leading to a decay of the average spin polarization.

The panels on top of the maps in Fig. SM6 display the average spin polarization ρ_s obtained by integrating the spin projection maps for $|y| < 2$ μm. The superimposed dashed lines are fits to Eq. (1) of the main text, which yield the effective SO length $L_{SO} = \Omega_{SO}/v_{\rm SAW}$ as well as spin decay length ℓ_s (see below). For the widest channel in Fig. SM6, the spin polarization decays with a distance ℓ_s corresponding roughly to $L_{\rm SO}^{(2D)}$. However, ℓ_s increases

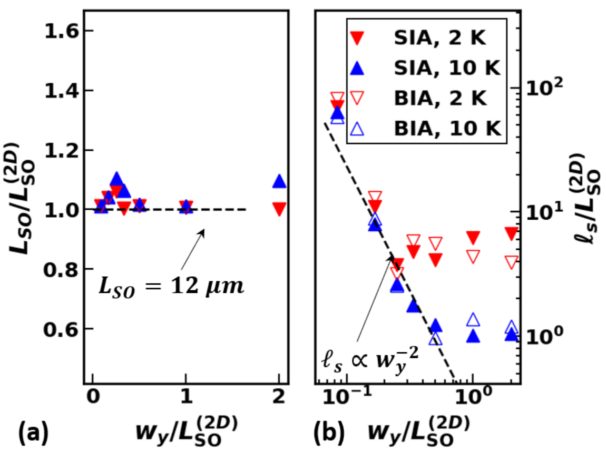

FIG. SM8. Dependence of the fitted (a) spin-orbit precession ratio $L_{SO}/L_{SO}^{(2D)}$ and (b) spin dephasing length (ℓ_s) normalized to the precession period $L_{SO}^{(2D)} = 2\pi v_{SAW}\Omega_{SO}$. The parameters were obtained from fits to Monte-Carlo simulations of the acoustic spin transport along channels with different widths w. Results are shown for transport at 2 K and 10 K under a BIA-type and SIA-type spin-orbit field. The L_{SO} and ℓ_s values were obtained by fitting the calculated spin polarization profiles to Eq. (1) of the main paper.

significantly with decreasing channel width, as shown in the upper plots of Figs. SM6(b) and SM6(c).

One interesting question is how the spin dynamics depends on the symmetry of the SO interaction. In order to address this question, Fig. SM7 displays spin polarization profiles calculated for different ratios r_s between the SIA and BIA SO contributions. The left and right panels illustrate the momentum dependence of the SO coupling for each of the cases. The calculations were carried out for different channel widths w_y. Figures SM7(a) and SM7(d) are for the pure BIA and SIA contributions, respectively. Within the calculation accuracy, one observes a similar behavior for the two types of asymmetry with the spin decay length ℓ_s increasing with decreasing channel width. Figures SM7(b) and SM7(c) show the simulation results for $r_s = 0.5$ and $r_s = -0.5$, which correspond to the spin helix modes with $\mathbf{\Omega}_{SO}$ parallel to y and x, respectively (cf. right panels) [24–26]. In both cases, the spin decay reduces significantly.

The dependence of the spin precession period (L_{SO}) and decay lengths obtained from fits of the calculated profiles to Eq. (1) of the main text are summarized in Figs. SM8(a) and (b), respectively. Results are presented for simulations under SIA (filled symbols) and BIA (open symbols) SO fields at the temperatures of 2 K and 10 K, which yield different carrier mean-free-path ℓ_p. The fitted precession periods are found to coincide within 10% with the period $L_{SO}^{(2D)} = 12$ μm quantifying the SO interaction strength in the simulations. Such a behavior is indeed expected from Eqs. (SM18) for $r_s > 0$, when the magnitude $|\Omega_{SO}|$ is constant. Since the time Δt for propagation over a short distance $\Delta r = (\Delta x, \Delta y)$, given by $\Delta t = \Delta r/v = m_e^*\Delta r/(\hbar k)$, is inversely proportional to k, then the precession angle around y can be expressed as $\delta\phi = \Omega_{SO_y}\Delta t = \Omega_{SO}\cos\theta\Delta t = (\hbar/m_e^*)\Omega_{SO}\Delta x$. As a consequence, the precession period along x does not depend on the spin propagation angle θ.

The dependence of ℓ_s on w_y displayed in Fig. SM8(b) shows two regions with different behaviors. For wide channels, ℓ_s saturates at a value

$$\ell_s \propto v_{SAW} * \frac{(L_{SO}^{(2D)})^2}{2\pi v_y^2 \tau_p}, \qquad \text{(SM22)}$$

which reduces with increasing temperature. For narrow channels, in contrasts, ℓ_s is essentially independent of temperature and increases according to $\ell_s \propto w^{-2}$. Such a dependence agrees with previous simulation results from Refs. 3 and 23 and is attributed to the increased role of motional narrowing for $w_y < L_{SO}^{(2D)}$.

The results of this section thus show that while the spin precession frequency during acoustic transport is essentially independent of the channel width, the spin decoherence lengths increase significantly in narrow channels ($w_y << L_{SO}$).

[1] B. Baylac, X. Marie, T. Amand, M. Brousseau, J. Barrau, and Y. Shekun, Hole spin relaxation in intrinsic quantum wells, Surf Sci **326**, 161 (1995).

[2] H. Sanada, T. Sogawa, H. Gotoh, K. Onomitsu, M. Kohda, J. Nitta, and P. V. Santos, Acoustically induced spin-orbit interactions revealed by two-dimensional imaging of spin transport in GaAs, Phys. Rev. Lett. **106**, 216602 (2011).

[3] A. A. Kiselev and K. W. Kim, Progressive suppression of spin relaxation in two-dimensional channels of finite width, Phys. Rev. B **61**, 13115 (2000).

[4] A. Hernández-Mínguez, K. Biermann, R. Hey, and P. V. Santos, Spin transport and spin manipulation in (110) and (111) GaAs quantum wells, Phys. Status Solidi B **251**, 1736 (2014).

[5] M. Z. Maialle, E. A. de Andrada e Silva, and L. J. Sham, Exciton spin dynamics in quantum wells, Phys. Rev. B **47**, 15776 (1993).

[6] M. I. Dyakonov, V. A. Marushchak, V. I. Perel, M. N. Stepanova, and A. N. Titkov, Spin relaxation of conduction electrons in uniaxial deformed crystal a_3b_5, Bull. Acad. Sci. USSR, Phys. Ser. **47**, 23 (1983).

[7] G. E. Pikus, V. A. Marushchak, and A. N. Titkov, Spin splitting of energy bands and spin relaxation of carriers in cubic III-V crystals (review), Sov. Phys. Semicond. **22**, 115 (1988).

[8] F. Meier and B. P. Zakharchenya, *Optical orientation*, edited by V. M. Agranovich and A. A. Maradudin, Mod-

ern problems in condensed matter physics No. 8 (North-Holland, Amsterdam, The Netherlands, 1984).

[9] M. Beck, C. Metzner, S. Malzer, and G. H. Doehler, Spin lifetimes and strain-controlled spin precession of drifting electrons in zinc blende type semiconductors, Europhys. Lett. **75**, 597 (2006).

[10] L. C. Lew Yan Voon and L. R. Ram-Mohan, Tight-binding representation of the optical matrix elements: Theory and applications, Phys. Rev. B **47**, 15500 (1993).

[11] J. N. Schulman and Y.-C. Chang, Band mixing in semiconductor superlattices, Phys. Rev. B **31**, 2056 (1985).

[12] M. Cardona, N. E. Christensen, and G. Fasol, Relativistic band structure and spin-orbit splitting of zinc-blende-type semiconductors, Phys. Rev. B **38**, 1806 (1988).

[13] J. C. Slater and G. F. Koster, Simplified lcao method for the periodic potential problem, Phys. Rev. **94**, 1498 (1954).

[14] P. V. Santos and M. Cardona, Comment on: Observation of spin precession in GaAs inversion layers using antilocalization, Phys. Rev. Lett. **72**, 432 (1994).

[15] P. V. Santos, M. Willartzen, M. Cardona, and A. Cantarero, Tight-binding calculations of spin splittings in semiconductor superlattices, Phys. Rev. B **51**, 5121 (1995).

[16] P. Vogl, H. P. Hjalmarson, and J. D. Dow, A semi-empirical tight-binding theory of the electronic structure of semiconductorsâ€ , J. Phys. Chem. Solids **44**, 365 (1983).

[17] K. C. Hass, H. Ehrenreich, and B. Velický, Electronic structure of $hg_{1-x}cd_x$Te, Phys. Rev. B **27**, 1088 (1983).

[18] D. J. Chadi, Spin-orbit splitting in crystalline and compositionally disordered semiconductors, Phys. Rev. B **16**, 790 (1977).

[19] G. E. Pikus and A. N. Titkov, Optical orientation (North Holland, Amsterdam, Amsterdam, 1984) Chap. Spin relaxation under optical orientation in semiconductors, p. 73.

[20] M. I. D'yakonov, V. A. Marushchak, V. I. Perel', and A. N. Titkov, The effects of strain on the spin relaxation of conduction electrons in III-V semiconductors, Sov. Phys. JETP **63**, 665 (1986).

[21] J. A. H. Stotz, R. Hey, P. V. Santos, and K. H. Ploog, Spin transport and manipulation by mobile potential dots in GaAs quantum wells, Physica E **32**, 446 (2006).

[22] M. M. de Lima, Jr. and P. V. Santos, Modulation of photonic structures by surface acoustic waves, Rep. Prog. Phys. **68**, 1639 (2005).

[23] A. A. Kiselev and K. W. Kim, Suppression of Dyakonov-Perel spin relaxation in 2d channels of finite width, physica status solidi (b) **221**, 491 (2000).

[24] B. A. Bernevig, J. Orenstein, and S.-C. Zhang, Exact SU(2) symmetry and persistent spin helix in a spin-orbit coupled system, Phys. Rev. Lett. **97**, 236601 (2006).

[25] J. D. Koralek, C. P. Weber, J. Orenstein, B. A. Bernevig, S.-C. Zhang, S. Mack, and D. D. Awschalom, Emergence of the persistent spin helix in semiconductor quantum wells, Nature **458**, 610 (2009).

[26] M. P. Walser, C. Reichl, W. Wegscheider, and G. Salis, Direct mapping of the formation of a persistent spin helix, Nat. Phys. **8**, 757 (2012).
